# Asymmetric and zwitterionic Blatter diradicals†

**DOI:** 10.1039/d3sc00367a

**Published:** 2023-02-21

**Authors:** Fang Miao, Yu Ji, Bo Han, Sergio Moles Quintero, Hanjiao Chen, Guodong Xue, Lulu Cai, Juan Casado, Yonghao Zheng

**Affiliations:** a Department of Pharmacy, Sichuan Provincial People's Hospital, University of Electronic Science and Technology of China Chengdu 610072 People's Republic of China zhengyonghao@uestc.edu.cn; b Chengdu University of Traditional Chinese Medicine State Key Laboratory Southwestern Chinese Medicine Resources Chengdu 611137 People's Republic of China hanbo@cdutcm.edu.cn; c Department of Physical Chemistry, University of Málaga, Campus de Teatinos s/n Málaga 29071 Spain casado@uma.es; d Analytical & Testing Center, Sichuan University Chengdu 610064 People's Republic of China; e School of Optoelectronic Science and Engineering, University of Electronic Science and Technology of China Chengdu 610054 People's Republic of China; f Institute of Electronic and Information Engineering of UESTC in Guangdong Zongbu Second Road No. 17 Dongguan Guangdong 523808 People's Republic of China

## Abstract

Asymmetric diradical molecular systems with different resonance mechanisms are largely unexplored. Herein, two conjugated asymmetric diradicals with Blatter and phenoxyl moieties (pBP and mBP) have been synthesized and studied in depth. A complete set of spectroscopic, X-ray crystallographic and magnetic techniques, together with quantum chemical calculations, have been used. The *para*-isomer (pBP) bears diradical and zwitterionic resonant forms, the latter by a electron delocalization mechanism, which are synergistically integrated by a sequence of nitrogen, provided by the Blatter moiety imine and amine (of different acceptor nature). In the *meta*-isomer (mBP), the zwitterionic form promoted in pBP by the lone-pair electron of the amine nitrogen is not available, yet it possesses a pseudo-hyperconjugation effect where the N lone pair mediates in a bonding coupling in a counter homolytic bond scission mechanism. Both electronic effects converge to promote medium diradical characters and narrow singlet–triplet gaps to the two electronic isomers. All these aspects delineate the subtle balance that shapes the electronic structure of open-shell molecules, which is even more challenging in the case of asymmetric systems, such as those described here with asymmetric phenoxyl–Blatter diradicals.

## Abstract 2

Asymmetric diradical molecular systems with different resonance mechanisms are largely unexplored. Two conjugated asymmetric diradicals with Blatter and phenoxyl moieties (pBP and mBP) have been synthesized and studied in depth.

## Graphical abstract

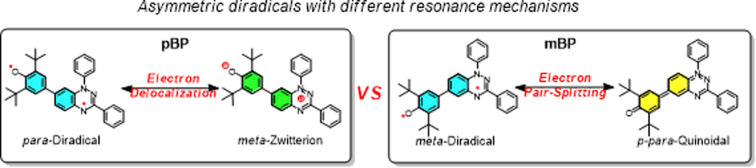

## 1 Introduction

Nowadays, open-shell molecules are promising components of next-generation molecule-based magnets^1^ for spintronics,^2^ and as conductors and semiconductors in organic electronics.^3^ Whereas spintronics requires molecules with tunable low-to-high spin transitions, for the purpose of conducting and photoconducting materials the requirement is for materials able to stabilize charge-separated states (zwitterion-like structures). The occurrence of charge-transfer processes in the excited states is also of relevance for the application of organic chromophores in efficient organic photovoltaics (for example, singlet exciton fission^3^ to produce multiple triplet excitons).

**Scheme 1:**
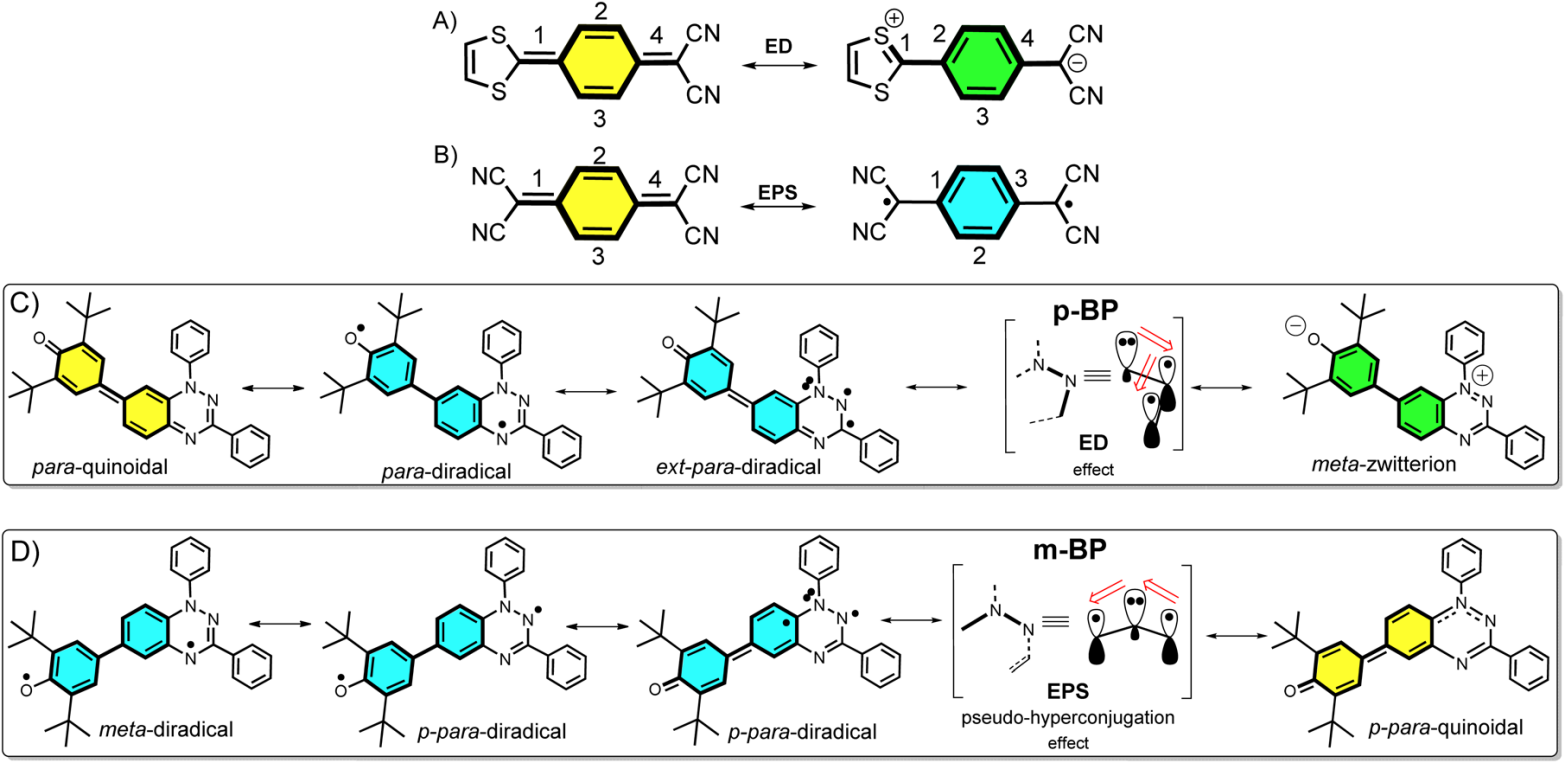
*Para*-disubstituted benzoquinoid ring that transforms into a zwitterion by electron delocalization (A, ED) and into a diradical by electron-pair splitting (B, EPS). ED resonant forms have the same number of double bonds, while the diradical *via* EPS has one bond less, leading to the dominance of A over B if both effects act on the same π path. (C) Resonance structures of pBP (in brackets the relevant π-conjugation center is highlighted with the amine N acting as donor). (D) Resonance structures in mBP (in brackets the relevant π-conjugation center is highlighted with the amine N acting as a transmitter of the inter-radical coupling).

Asymmetric diamagnetic π-conjugated zwitterions made by pro-aromatic spacers substituted with electron donor–acceptor groups (Scheme 1A) have been designed, historically, for electro-optical organic applications, such as in non-linear optics. The electronic property of relevance is the appearance of an electron delocalization effect (ED, Scheme 1A) by means of an intramolecular charge transfer that boosts the linear and non-linear optical responses. On the other hand, symmetric paramagnetic π-conjugated neutral diradicals based on quinonoid molecules^4^ are capable of producing neutral diradicals with low energy lying, high-spin triplet states, thanks to the gain of aromaticity in the bridge rings by means of an electron-pair splitting effect (EPS, Scheme 1B).

The majority of known π-conjugated diradicals^5^ and asymmetric zwitterions^6^ have been designed by substitution in the relative *para*-positions of the π-conjugated bridges (*i.e.*, 1,4-disubstitution of the central benzene in Scheme 1A and B) with radical-bearing moieties or with donor–acceptor electroactive groups, respectively. The two concepts have thus been independently explored. It turns out, however, that the number of molecules that simultaneously execute both ED and EPS effects over the same π-core is limited, and to some extent has been disregarded. As a result, the attainment of diradicals enhanced with asymmetric zwitterionic forms is challenging, with the main difficulty being in the ability to balance both ED and EPS through bond effects (*i.e.*, these effects are competitive in the sense that one increases to the detriment of the other). This situation is described in Scheme 1A and B, where it can be realized that, in principle, the diradical structure is disfavored with respect to the zwitterionic structure, as the former always has a smaller number of double bonds in conjugation. Hence, diradicals with charge-transfer character, or zwitterions with accessible high-spin states, are uncommon and achieving them is of interest. Some attempts along these lines have been made, such as that by Wu *et al.*^7^ with a series of conjugated push–pull diradicals based on quinoidal perylenes. Other cases of asymmetric donor–acceptor diradicals are those in which the radical–radical and donor–acceptor groups are connected by means of isolating non-conjugated bridges, thus neglecting the mutual benefit provided by balancing the ED and EPS effects.^8^

Structural isomerism^9^ plays an important role in diversifying the electronic and magnetic properties of molecules. For example, in disubstituted benzenes, when the isomerization pattern of the two substituting radical centers with regard to the central bridge changes from *para* to *meta*, the diradical character is increased and the ground electronic state can even vary from singlet to triplet.^10^

In this article, we attempt to implement the ED and EPS effects by synthesizing two asymmetric diradical isomers (pBP and mBP in Scheme 1) made by phenoxyl radical (acceptor) and Blatter radical (donor) moieties. The objective is to formulate new examples of zwitterion–diradical structures. With respect to the central benzene (Blatter benzene) of the pBP and mBP molecules, the phenoxyl oxygen and iminyl nitrogen (of the other triazine Blatter ring) are disposed in relative *para*-positions in pBP and in *meta*-positions in mBP (Scheme 1). This dual pattern of substitution gives rise to an amalgam of distinctive canonical and resonant forms in asymmetric pBP and mBP, producing in both cases charge-transfer diradicals or high-spin zwitterions, with, at the same time, some modulation from one to the other.

## 2 Results and discussion

### 2.1 Synthesis

**Scheme 2:**
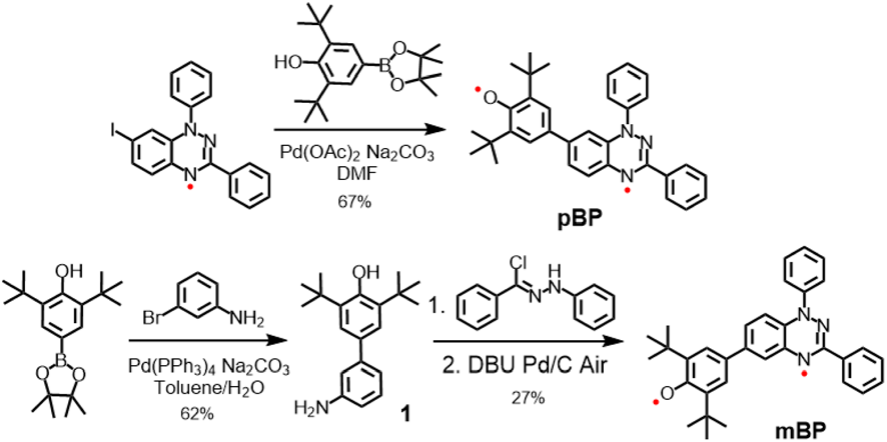
Synthetic routes for pBP and mBP.

Considering the good stability and facile syntheses of Blatter radicals^11^ and phenoxyl radicals,^12^ we proposed the synthesis of asymmetric diradical isomers with these two types of scaffolds, as displayed in Scheme 2. Compound pBP was synthesized directly from 7-iodobenzotriazinyl through a modified Suzuki reaction, with an acceptable yield. pBP is very stable and can be purified by column chromatography on triethylamine treated with silica gel under ambient conditions. On the other hand, amino-substituted compound 1 was obtained from a Suzuki coupling reaction. Then, amino 1 and *N*-phenyl-benzenecarbohydrazonoyl chloride provided amidrazone. Subsequently, amidrazone was subjected to reductive cyclization to mBP, following an established route.^13^ The two isomers were fully characterized by electron spin resonance (ESR) spectroscopy, superconducting quantum interference device (SQUID) measurement, single-crystal X-ray diffraction (XRD), UV-visible–near-infrared (NIR), Raman and Fourier-transform infrared (FTIR) spectroscopies.

### 2.2 Molecular structure from X-ray diffraction

**Fig. 1:**
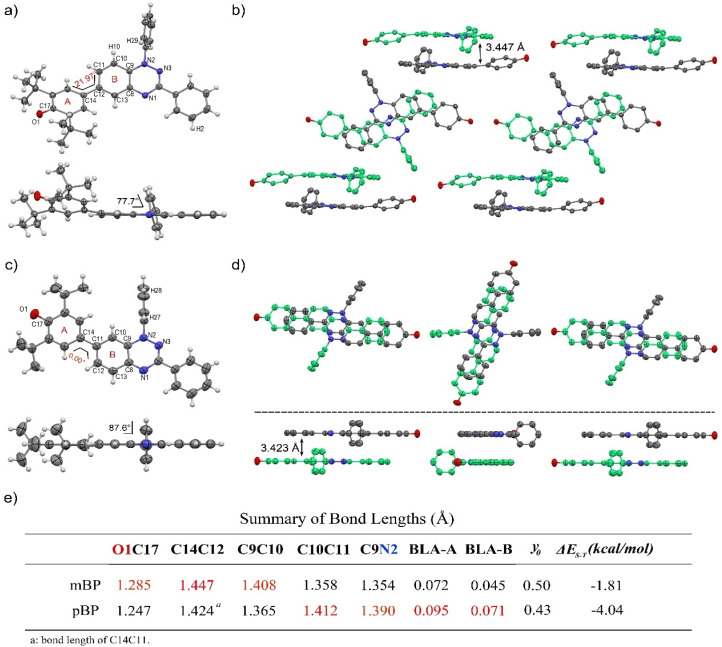
Single-crystal structures of mBP (a) pBP (c) and packing diagrams of mBP (b) and pBP (d). Thermal ellipsoids are drawn at 50% probability. Hydrogen atoms and *tert*-butyl groups are omitted for clarity. Dihedral angles, bond lengths (Å) and short contacts (Å) are labeled. (e) Summary of bond lengths and BLA of rings A and B.

Single crystals of pBP and mBP suitable for X-ray diffraction were obtained by recrystallization from dichloromethane/hexane. As shown in Fig. 1, the phenoxyl and benzotriazinyl moieties displayed dihedral angles of 0° and 21.97° in pBP and mBP, respectively. Simultaneously, we found that: (1) the distance of the CC bond between ring A and B in Fig. 1 for pBP (1.424 Å) is shorter than that of mBP (1.447 Å), and (2) the CO bond distances are 1.247 and 1.285 Å for pBP and mBP, respectively. All these distinctive features of pBP in relation to mBP are due to the larger contribution of the quinoid closed-shell form in pBP (Scheme 1C, *para*-quinoidal). Despite this superior quinoidal contribution in pBP, a significant amount of diradical character persists, as deduced from the similarity of its CO bond distance with that of typical monophenoxyl radicals (1.248 Å).^14^ In contrast, the CN distance (C9N2 in Fig. 1) of pBP is large, 1.390 Å, revealing limited electronic interaction through this bond towards the amino nitrogen. The situation is reversed in mBP where the more distorted structure is accompanied by a CN (C9N2) bond distance that is shorter (1.354 Å) than in pBP, in agreement with the stronger electron delocalization over this bond. Using quantum chemical calculations, the bond length alternations of rings A and B of pBP and mBP were calculated (Fig. 1e) and display a larger value for pBP, in accord with the larger role of its quinoidal form.

The supramolecular structure of the mBP crystal comprises a centrosymmetric dimer linked through a pair of equivalent CH⋯N contacts (N1H29) between a triazinyl N (at position N1) and an *ortho*-hydrogen atom of the *N*-phenyl ring. The distance between dimer molecules is 3.447 Å (Fig. 1b). On the other hand, the pBP molecule has a symmetry plane and forms one-dimensional stacked columns with distances of 3.423 Å, which are also assembled through the *ortho* CH⋯N interactions (N1H27, Fig. 1d). Interestingly, both distances are shorter than that in the Blatter radical (3.45 Å),^15^ which indicates that the phenoxyl substitution could enhance the intermolecular interaction. Finally, the π-dimers are formed confronting the phenoxyl and Blatter moieties, as are typically found in donor–acceptor systems by interaction of fragments with charges of different signs.^16^

### 2.3 Magnetic properties

The magnetic properties of both compounds were investigated by variable-temperature ESR measurements in powder form. The intensity (*I* × *T*) of the double integral ESR intensity (*I*) multiplied by temperature (*T* in K) decreased upon cooling (Fig. S1†), suggesting depopulation of the high-spin state in favor of a low-spin ground state, or triplet to singlet transition, on cooling. From fitting of this curve with the Bleaney–Bowers equation, the energy difference between the singlet (*E*_S_) and triplet states (*E*_T_), Δ*E*_ST_ (2*J*), was estimated, and amounted to −1.01 and −1.71 kcal mol^−1^ for mBP and pBP, respectively. Moreover, we have carried out the same variable-temperature ESR experiment in dilute solid solution (benzoquinone glassy matrices with concentration of 0.1 mM) to avoid the effect of intermolecular interactions on the magnetic properties. We found that the Δ*E*_ST_ values were similar in dilute solution and in solid powder conditions (Fig. S2†). These Δ*E*_ST_ values are well reproduced by quantum chemical calculations, at −1.81 and −4.04 kcal mol^−1^, respectively. The quantum chemical quantity related to the diradical character,^17^*y*_0_ (which is *y*_0_ = 0 for closed-shell systems and *y*_0_ = 1 for full open-shell diradicals), has also been estimated, giving *y*_0_ = 0.50 for mBP and *y*_0_ = 0.43 for pBP. In mBP the phenoxyl and iminyl radical are placed at the *meta*-positions of the central benzene bridge, a situation that typically produces triplet ground electronic states in benzene *meta*-disubstituted diradicals. However, the *y*_0_ = 0.50 in the singlet diradical ground electronic state for mBP, compared to *y*_0_ = 1 as would be expected for a *meta*-diradical triplet, indicates substantial coupling of the unpaired electrons, which takes place through the *p-para*-quinoidal path shown in Scheme 1D. It is noticeable that the two molecules share similar values of diradical characters in spite of their different isomeric patterns: (1) in pBP, the singlet ground electronic state is contributed by the quinoidal and zwitterionic structures, resulting in *y*_0_ = 0.43; whereas, (2) in mBP, the *y*_0_ = 0.50 singlet diradical character is imparted by the inter-radical bonding through the amine N along the *para*-conjugated path (Scheme 1D).

**Fig. 2:**
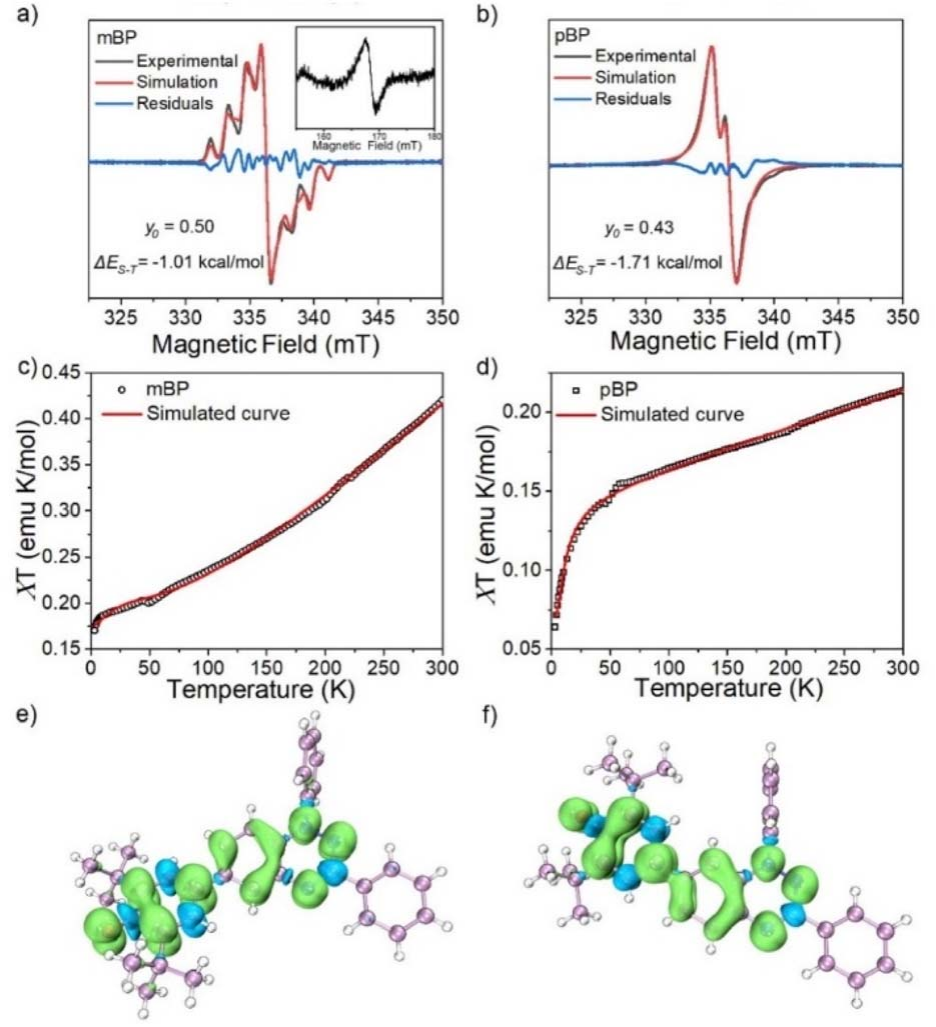
ESR spectra of mBP (a) and pBP (b) in toluene, at 130 and 140 K, respectively. The black, red and blue lines indicate experimental, simulated and difference ESR (experimental-simulated) spectra, respectively. The inset shows the half-field ESR signal at 130 K. Magnetic susceptibilities (*χT*) *versus T* curve from the SQUID measurements on the powder of mBP (c) and pBP (d) and the fitting plot obtained with the Bleaney–Bowers equation. Calculated spin density of mBP (e) and pBP (f) based on single-crystal structure at the (u)m062x/6-311(d,p) level.

The small Δ*E*_ST_ values obtained for the two compounds allow facile thermal population of the lowest lying triplet excited states, which agrees with the observation of a half-field forbidden transition signal (Δ*m*_S_ = ±2) for mBP. No half-field forbidden transition signal was found for pBP. Additional evidence for the presence of triplet states in the two compounds is that their ESR spectra in toluene (Fig. 2a and b) show characteristic triplet patterns at low temperature. From simulations of the ESR spectra of the triplet of mBP, the resulting ZFS parameters are *D* = 0.0029 cm^−1^ and *E* = 0.00056 cm^−1^, together with an average distance between the two spin centers of 9.64 Å, according to the point dipole limit. This distance is longer than that measured between the oxygen and the imine nitrogen of mBP (8.76 Å) from the single-crystal structure, in line with the role of the *p-para*-quinoidal form in Scheme 1D.

The magnetic behavior of mBP and pBP in powder forms and in dilute solid solution (benzoquinone glassy matrices with concentration of 40 mM) were studied by measuring magnetic susceptibilities (*χT*) using SQUID in the temperature range 2–300 K and with an applied field of 1.0 T (Fig. 2c and d). The data were corrected for both sample diamagnetism (Pascal's constants, −0.000269 emu mol^−1^) and the diamagnetism of the sample holder (polycarbonate capsule) and benzoquinone. The measured *χT*–*T* can be fitted with the modified Bleaney–Bowers equation. The magnetic susceptibility behavior of the compounds is coherent with antiferromagnetic interactions between the unpaired electrons in the singlet diradical state, whose triplet state is populated by the thermal excitation. In addition, the majority of the calculated spin densities (*α* minus *β* spin densities) of the two isomers is spread across the phenoxyl and Blatter moieties (Fig. 2e and f).

### 2.4 Spectroscpic properties

**Fig. 3:**
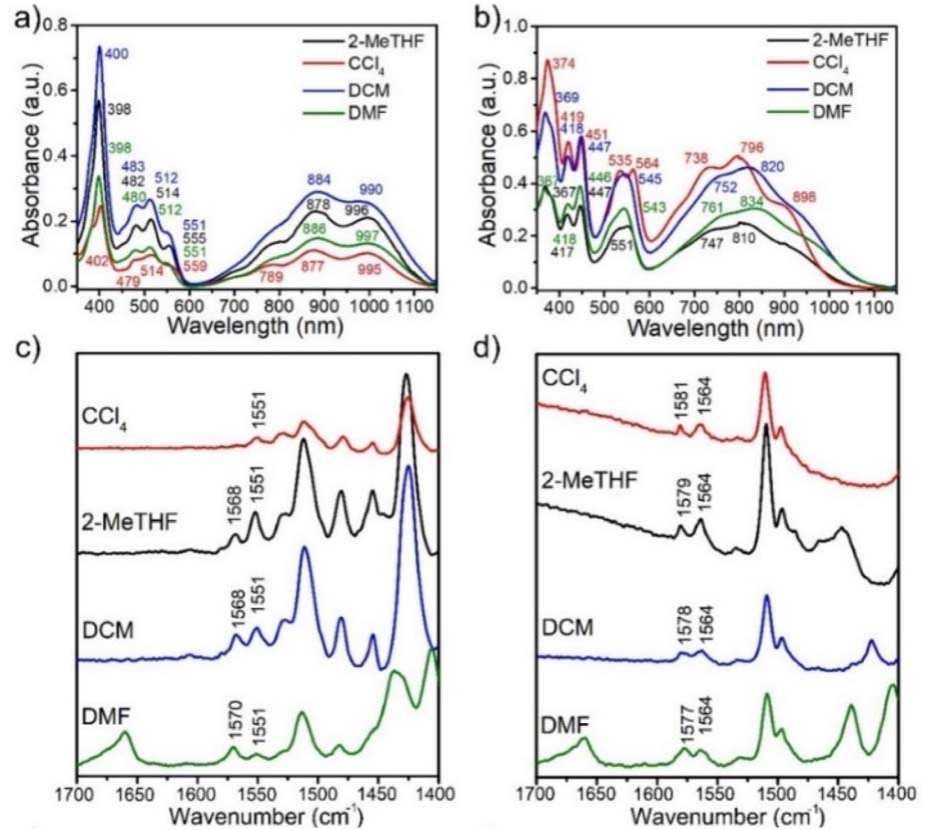
UV-vis–NIR electronic absorption spectra of mBP (a) and pBP (b) in solvent of different polarity (green: DMF, blue: dichloromethane (DCM), red: CCl_4_, and dark: 2-methyltetrahydrofuran (2-MeTHF)). Solution-state 785 nm excitation Raman spectra of mBP (c) and pBP (d) in several solvents.

The zwitterionic character of the mBP and pBP isomers has also been investigated by electronic absorption and vibrational Raman spectroscopies. Both compounds display absorption bands in the NIR spectrum region, in line with the typical spectra of diradicaloids. Given the analyzed contributions of the zwitterionic forms, solvatochromism in solvents of different polarity has been measured, such as that displayed in Fig. 3. The wavelength of the maxima of the lowest energy absorptions of pBP displays a hypsochromic shift from dimethylformide (DMF) to tetrachloromethane (CCl_4_) of 38 nm (−620 cm^−1^), while this shift is only 8 nm (−102 cm^−1^) in mBP, highlighting the marked negative solvatochromism^18^ in the two compounds. Negative solvatochromism might be ascribed to the zwitterionic contribution in the ground electronic state of pBP in Scheme 1C. On the other hand, the EPS interaction through the *p-para*-quinoidal path between the phenoxyl oxygen and the imine Blatter N of mBP, which causes the reduction of *y*_0_ from 1 to 0.5, might also originate from a certain donor–acceptor polarization with subsequent modest negative solvatochromism. Overall, this reflects the more efficient establishment of a charge-separated zwitterionic state in pBP, whereas only an incipient polarized state through the *p-para*-quinoidal form is deduced in mBP.

Fig. 3 displays the Raman spectra of mBP and pBP in the same solvents. In almost all cases, the 785 nm Raman spectra of mBP show a pair of bands in the 1580–1550 cm^−1^ region due to CC stretching modes of the Blatter and of the phenoxyl moieties. pBP shows this pair of bands always at higher wavenumbers than mBP, in agreement with the contribution of a quinoidal resonant form that, as seen previously, enlarges the bond length alternation, and consequently its associated vibrational frequencies. For pBP, the 1581 cm^−1^ band in CCl_4_ moves to 1575 cm^−1^ in DMF, while for mBP the 1570 cm^−1^ band scarcely moves, by 2 cm^−1^, in the studied solvents. The 1581 → 1575 cm^−1^ shift in pBP reveals the ground electronic state contribution to the negative solvatochromic effect.

### 2.5 Electronic structure and operating mechanisms

**Fig. 4:**
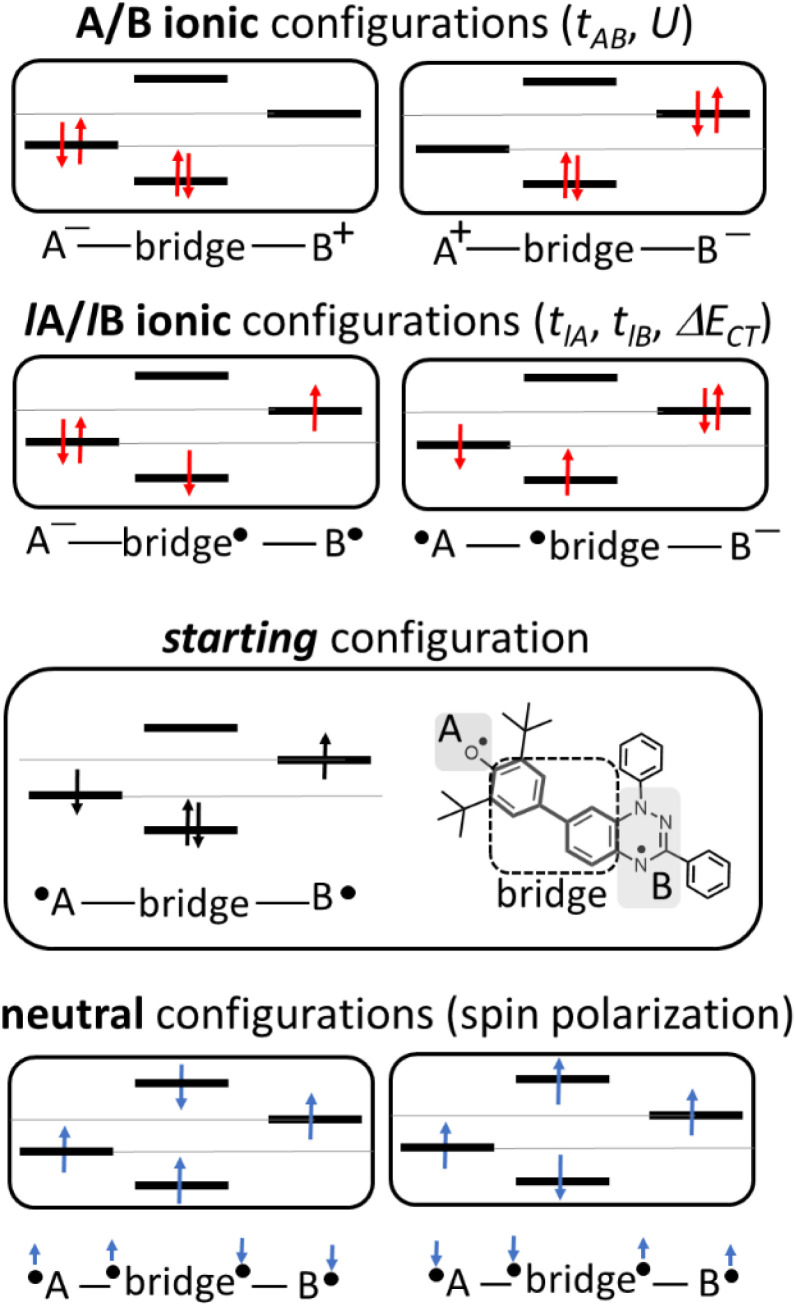
Electronic configurations for the asymmetric diradicals studied here, where A and B are the phenoxyl oxygen and imine nitrogen in the case of pBP: ionic forms (top) and neutral tetra-radical forms (bottom).

In the two-electron two-site model^19^ (A and B in Fig. 4) and within the second-order perturbation theory valence configuration interaction approach, the singlet–triplet gap, Δ*E*_ST_, is obtained in terms of: (1) an exchange ferromagnetic interaction (direct exchange, *K*_AB_, related to the direct overlap between sites A and B); (2) a transfer integral (*t*) between sites A and B; and (3) an energy difference between the on-site and inter-site electron repulsions (*U*) according to the following expression for symmetric diradicals (A and B are identical):
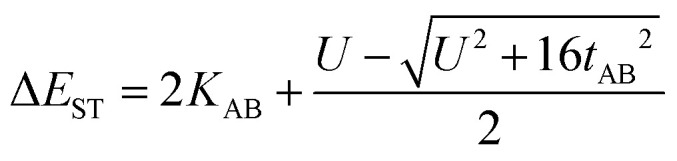


This expression can be simplified by considering that *U* ≫ |*t*_AB_|, which is reasonable in our systems given the moderate-to-large diradical character (note that *U* ≪ |*t*_AB_| is the usual situation in closed-shell systems), by which the above expression for symmetric diradicals transforms to:
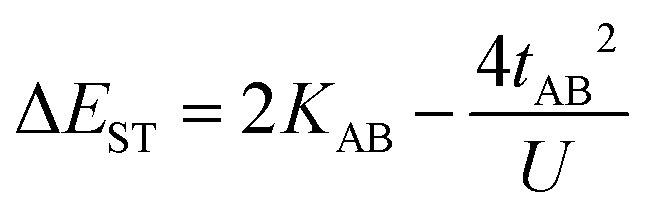


Which accounts for the presence of a ferromagnetic (*K*_AB_) and an antiferromagnetic term 
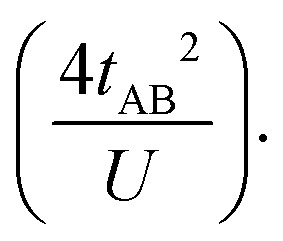
 The last adaptation of this formula to our case should consider the asymmetric character of the studied diradicals, which mostly affects the antiferromagnetic part by means of the transfer integrals. Hence, the above expression for asymmetric diradicals becomes:
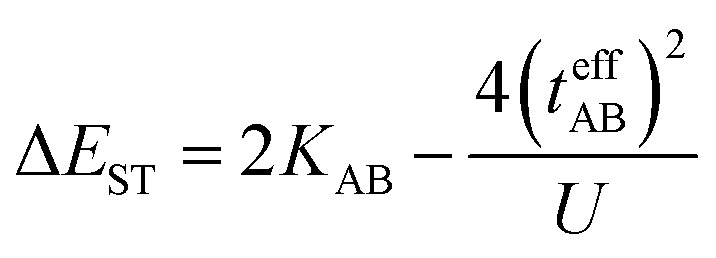
where
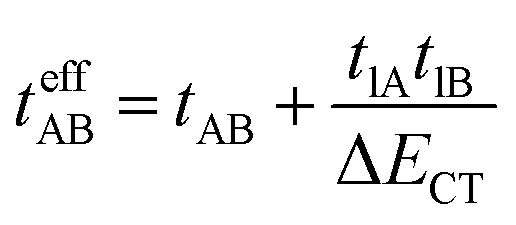
where *t*_lA_ and *t*_lB_ denote the transfer integral interactions between the bridge (designated as l and located between the radical sites A and B) and sites A (*t*_lA_) and B (*t*_lB_). Finally, Δ*E*_CT_ provides the energy gap perturbative correction for the interaction between the bridge and the two A/B sites. Fig. 4 depicts the main electronic configurations of our asymmetric diradicals, which will serve us in dissecting two important pieces of information in this Δ*E*_ST_ description. First, the hopping integral (*t*) is the quantum mechanical quantity that mixes the singlet closed-shell ionic excited configurations with the diradical open-shell form (starting form in Fig. 4), which contributes to the antiferromagnetic coupling of the two radicals.^20^ The increasing weight or mixing with the ionic configurations (*i.e.*, larger *t*) makes the diradical character decrease and the Δ*E*_ST_ increase. On the other hand, the double spin-polarization effect^21^ is a through-bond bridge-mediated mechanism that tunes the Δ*E*_ST_ by stabilizing the singlet due to local reduction of the repulsion by spin polarization between the paired electrons of the bridge and of the radical centers. Double spin polarization accounts for the neutral electronic configurations of the type ˙A-˙bridge˙-B˙ in Fig. 4. Both mechanisms, ionic mixing and spin polarization, contribute to the modulation of Δ*E*_ST_, which will be addressed below in the case of the asymmetric isomers mBP and pBP.

According to Fig. 4, for pBP, the A site is the oxygen/phenoxyl and the B site is the imine N (N1)/Blatter, which both have different electron-acceptor character. For mBP, the B site is an amine N, which is endowed with donor character, and consequently the Δ*E*_CT_ and *U* terms are different in mBP compared with pBP. On the other hand, for *t*_AB_ and *t*_lB_, larger values are expected for pBP given that the sp^2^ character of oxygen and of the N atoms, which both interact through the *para*-positions, would favor overlap with and through the phenyl bridge, providing significant transfer integrals that would reduce the diradical character. Conversely, the O˙ → N˙ coupling occurs through *meta*-positions in mBP, by which the resulting overlap with the bridge is significantly minimized. Another situation is the possibility of a zwitterionic ED from the amine N of mBP up to the phenoxyl oxygen (feasible through the *para*-positions), which would mediate in the transmission of the O˙ → N˙ coupling (Scheme 1D), thus increasing the *t*_lA_ and *t*_lB_ antiferromagnetic terms and concomitantly the weight of the ionic forms, thus overall decreasing the diradical character. The similarity of the *y*_0_ and Δ*E*_ST_ values found experimentally and theoretically for the two compounds might thus be accounted for the possibility of delocalizing the spin–spin coupling between the phenoxyl oxygen and the sp^2^ aminyl N radical through the *para*-position in mBP, opening up a “direct“ O˙(phenoxyl) → N˙(iminyl) interation. This electronic effect of spin–spin coupling is assisted by the lone-pair electron of the amine N, an effect that can be viewed as a result of the partial hybridization of this non-bonding orbital, which acquires bonding character in order to transmit the O˙(phenoxyl) → N˙(iminyl) coupling. This reveals a similar effect to the well-known effect of hyperconjugation,^22^ in the sense that conjugation acts through non-*p*_*z*_ carbon orbitals.^23^ This pseudo-hyperconjugative effect through the lone-pair electron of mBP apparently compensates the deficient π-mesomeric effect between the O˙(phenoxyl) → N˙ through the *meta*-positions of the bridge.

Finally, taking into consideration the tetraradical neutral configuration, ˙A-˙bridge˙-B˙, directly connected with the double spin-polarization mechanism, we realize that in mBP, double spin polarization would be inefficient because of the disconnection through the *meta*-disposition of the unpaired electrons relative to the bridge. However, this would again be compensated by the feasible interaction mediated by the amine N through pseudo-hyperconjugation.

## 3 Conclusions

We have designed and synthesized two asymmetric isomers (mBP and pBP) made of a phenoxyl group and a Blatter radical moiety. Both molecules constitute one example of open-shell, singlet ground electronic state asymmetric diradicals with medium diradical character and rather narrow singlet–triplet gaps. The isomerisation of the O˙ → N˙ connection in pBP and mBP of direct O˙ → N˙ cascades gives rise to different resonant forms. In particular, the spin–spin coupling effect in mBP, through the lone-pair electron of its amine N, and the zwitterionic structures in pBP play distinctive roles. This is confirmed by the realization of singlet ground electronic states and the measurement of small singlet–triplet energy gaps, together with solvatochromism.

While symmetric diradicals have proliferated in the recent literature, achieving conjugated asymmetric diradicals is less common, due to the very delicate balance between the donor–acceptor character, which annihilates the diradical character in favor of closed-shell zwitterions.^24^ This adverse situation has been saved here by incorporating an oxygen acceptor and a nitrogen donor with narrow donor–acceptor orbital energy gradient; thus, pBP is a new asymmetric diradical with a combination of zwitterion and diradical features. In the analogous isomer, mBP, the amine N lone-pair electron, instead of acting as an electronic barrier or stopper, turned out to behave as a good conjugation transmitter. This investigation delineates subtle aspects in the electronic structure of open-shell molecules and contributes to the formulation of a strategy for the design of molecules with magnetic properties in conjunction with zwitterions, which is potentially valid for the integration of molecule-based spintronics and electronics.

## Data availability

NMR spectra, mass spectra, Fourier transform infrared (FT-IR) spectra, ESR spectra, SQUID spectra, low temperature absorption spectra, cyclic voltammogram, thermogravimetric analysis, electrostatic potential surface and X-ray single crystal data can be found in the ESI.†

## Author contributions

Y. Z. supervised the project. F. M. carried out the synthetic work. H. C. and B. H. carried out the ESR studies. F. M., Y. J., L. C. and J. C. prepared the manuscript. S. M. Q. contributed to the Raman studies. G. X. and J. C. carried out the computational studies. All authors discussed the results and commented on the manuscript.

## Conflicts of interest

The authors declare no competing financial interest.

## Supplementary Material

SC-014-D3SC00367A-s001

SC-014-D3SC00367A-s002
