# Supplementary material for: Asymmetric and zwitterionic Blatter diradicals
Source: Chem Sci. 2023 Feb 21;14(10):2698–705. doi: 10.1039/d3sc00367a (PMC9993846; doi:10.1039/d3sc00367a)
Supplement: SC-014-D3SC00367A-s001 [file SC-014-D3SC00367A-s001.pdf]

Electronic Supplementary Material (ESI) for Chemical Science.
This journal is © The Royal Society of Chemistry 2023

Supporting Information

Asymmetric & Zwitterionic Blatter Diradicals

Fang Miao,[a,f†] Yu Ji,[a,e†] Bo Han,*[b] Sergio Moles Quintero,[c] Hanjiao Chen,[d†] Guodong Xue,[e] Lulu Cai,[a] Juan Casado,*[c] Yonghao Zheng*[a,e]

a. Department of Pharmacy, Sichuan Provincial People's Hospital, School of Optoelectronic Science and Engineering, University of Electronic Science and Technology of China, Chengdu 610072, People's Republic of China.
b. Chengdu University of Traditional Chinese Medicine, State Key Laboratory Southwestern Chinese Medicine Resources, Chengdu 611137, People's Republic of China.
c. Department of Physical Chemistry, University of Málaga, Campus de Teatinos s/n, Málaga 29071, Spain.
d. Analytical & Testing Center, Sichuan University, Chengdu 610064, People's Republic of China.
e. School of Optoelectronic Science and Engineering, University of Electronic Science and Technology of China, Chengdu 610054, People's Republic of China.
f. Institute of Electronic and Information Engineering of UESTC in Guangdong, Zongbu Second Road No. 17, Dongguan, Guangdong 523808, People's Republic of China.

E-mail addresses: zhengyonghao@uestc.edu.cn; hanbo@cdutcm.edu.cn; casado@uma.es;
† These authors contributed equally to this work.

1. Methods and Materials

All reagents were purchased from Sigma-Aldrich, Alfa, Acros and Adamas and used as received. Flash column chromatography was performed with Haiyang silica gel (200-300 mesh). Solvent toluene was freshly distilled from CaH_2 under N_2. All reaction mixtures and column eluents were monitored by TLC using commercial Huanghai glass plates (HSGF 254, 2.5 x 8 cm). The plates were visualized under UV radiation at 254 and 365 nm. ^1H and ^{13}C NMR spectra were recorded on a Bruker AV III HD 400 MHz. ESR measurements were carried out on a Bruker EMX plus X-band spectrometer with 9.8 GHz microwave frequency. High resolution mass spectra (HRMS) were measured on a Waters-Q-TOF-Premier (ESI). Single Crystal X-Ray Diffraction were measured by a Gemini X-ray Single Crystal Diffractometer. Thermogravimetric analysis (TGA) measurements were performed on NETZSCH TG 209F1 Iris thermal gravimetric analyzer. Cyclic voltammograms were measured on a Shanghai Chenhua CHI 660E electrochemical workstation in dry DCM with 0.1 M tetrabutylammonium hexafluorophosphate ($TBAPF_6$) as the supporting electrolyte at a scan rate of 100 mV/s at room temperature under the protection of nitrogen. A gold disk was used as working electrode, platinum wire was used as counter electrode, and Ag/AgCl (3 M KCl solution) was used as reference electrode. The potential was externally calibrated after each experiment, against the ferrocene/ferrocenium couple.

SQUID measurements were carried out on a Quantum Design (MPMS-SQUID VSM-094). Magnetic susceptibility of powder samples was measured in a polycarbonate capsule fitted in a plastic straw as a function of temperature in heating (2 K–300 K) mode with 30 seconds of temperature stability at each temperature (1 K increment in a range 2–10 K, 3 K increment in a range 10–300 K,) at 1.0 T using a SQUID magnetometer. The data was corrected for both sample diamagnetism (Pascal′s constants) and the diamagnetism of the sample holder (polycarbonate capsule). The singlet-triplet energy gap was estimated by fitting the I*T vs. T curve with modified Bleaney-Bowers equation[1,2].

All calculations were performed with the Gaussian 16 program suite[3] using the density functional theory (DFT) with M06-2X exchange-correlational functionals[4] and employing the 6-311G(d,p) basis set[5-7] for all atoms. Full geometry optimizations were carried out at the M06-2X/6–311G(d,p) level, and the obtained stationary points were characterized by frequency calculations. The spin

density distribution and electrostatic potential surface were illustrated using Multiwfn[8] and VMD[9].

The diradical character y_0 is calculated by Yamaguchi's equation based natural orbital analysis for the optimized singlet geometry:

$$y_0 = 1 - \left(2T/\left(1 + T^2\right)\right)$$

where T is represented by calculating the occupation numbers of natural orbitals:

$$T = (\text{nHONO} - \text{nLUNO})/2$$

A molecule with $y_0 = 0$ implies a closed-shell structure, whereas a molecule with $y_0 = 1$ indicates a pure diradical structure[10-11]. Any intermediate value of y_0 refers to diradical structures. The ΔE_{ST} were calculated using zero-point vibrational energy correct singlet-triplet energy gaps.

2. Detailed synthetic procedures

Compound **pBP**: 2,6-di-tert-butyl-4-(4,4,5, 5-tetramethyl-1,3,2-dioxaborolan-2-yl)phenol (66 mg, 0.20 mmol), 7-iodobenzotriazinyl (41 mg, 0.10 mmol), Na_2CO_3 (31.8 mg, 0.30 mmol), and ($Pd(OAc)_2$, 2.3 mg, 0.01 mmol) were dissolved in toluene and water (15 mL, 3mL) under nitrogen and then the reaction mixture was heated to reflux for one hour. Then, the reaction mixture was cooled to room temperature, washed with water, and extracted with DCM. The organic phases were dried and concentrated under reduced pressure. The organic solvent was removed under reduced pressure and the residue was purified by silica gel column chromatography (SiO_2 passivated with 1% Et_3N, hexanes: DCM = 1:1, Rf value of 0.5) and give compound **pBP** as a brownish solid (30 mg, 62% yield). Compound **pBP** is NMR silent. Thus, ^{1}H NMR and ^{13}C NMR spectra are not given. HR-MS (ESI): m/z calcd for $C_{27}H_{30}N_3O$: 487.2624; found, 488.2641 ($[M+H]^+$). m.p. 164-165 °C. IR (FTIR): 3064, 1560, 1528, 1513, 1493, 1384, 1307, 1278, 1094, 1036, 861, 829, 685, 619, 525 cm^{-1}.

Compound **1**: 2,6-di-tert-butyl-4-(4,4,5,5-tetramethyl-1,3,2-dioxaborolan-2-yl)phenol (332 mg, 1.0 mmol), 3-bromoaniline (344 mg, 2.0 mmol), Na_2CO_3 (222 mg, 2.0 mmol), and Tetrakis-(triphenylphosphine)palladium(0) ($Pd(PPh_3)_4$, 11.2 mg, 0.05 mmol) were dissolved in toluene and water (10 mL/ 2 mL) under nitrogen and then the reaction mixture was heated to reflux for 12 hours. Then, the reaction mixture was cooled to room temperature, washed with water, and extracted with DCM. The organic phases were dried and removed under reduced pressure, then the residue was purified by silica gel column chromatography (hexanes: DCM = 1:1, Rf value of 0.4) and give compound **1** as a brownish solid (181 mg, 61 % yield). ^{1}H NMR (400 MHz, $CDCl_3$): δ (ppm) 7.37 (s, 2H), 7.21 (t, J = 8 Hz, 1H), 6.97 (d, J = 8 Hz, 1H), 6.68 (dd, J_1 = 8.0 Hz, 1H), 5.24 (s, 1H), 1.49 (s, 18H). 13C-NMR (100 MHz, CD_2Cl_2): δ (ppm) 153.7, 145.9, 143.8, 136.2, 132.8, 129.8, 124.2, 118.4, 114.4, 113.9, 34.7, 30.6. HR-MS (ESI): m/z calcd for $C_{20}H_{27}NO$: 297.2091; found, 298.2170 ($[M+H]^+$).

Compound **mBP**: compound **1** (297 mg, 1.0 mmol), (E/Z)-N'-phenylbenzohydrazonoyl chloride (253 mg, 1.1 mmol), and triethylamine (151 mg, 1.5 mmol) were dissolved in toluene under nitrogen and then the reaction mixture was reacted at room temperature for 12 hours with white

precipitate appeared. Then, the reaction mixture was filtered. The filtrate was evaporated under reduced pressure, then the residue was treated with Pd/C (1.6 mol%) and 1,8-Diazabicycloundec-7-ene (DBU) (0.5 mL) in DCM. The reaction mixture was stirred in air at room temperature until a new brown precipitate appeared. This precipitate was filtered and purified by silica gel column chromatography (SiO_2 passivated with 1% Et_3N, hexanes: DCM = 1:1, Rf value of 0.6) and give compound **mBP** as a brownish solid (136 mg, 27 % yield). Compound **mBP** is NMR silent. Thus, ^{1}H NMR and ^{13}C NMR spectra are not given. HR-MS (ESI): m/z calcd for $C_{33}H_{33}N_3O$: 487.2624; found, 488.2641 ($[M+H]^+$). m.p. 178-179 °C. IR (FTIR): 3064, 1260, 1095, 1021, 801 cm^{-1}.

3. ESR spectra

Simulations of ESR spectra were performed with the *Easyspin* program in Matlab[12].

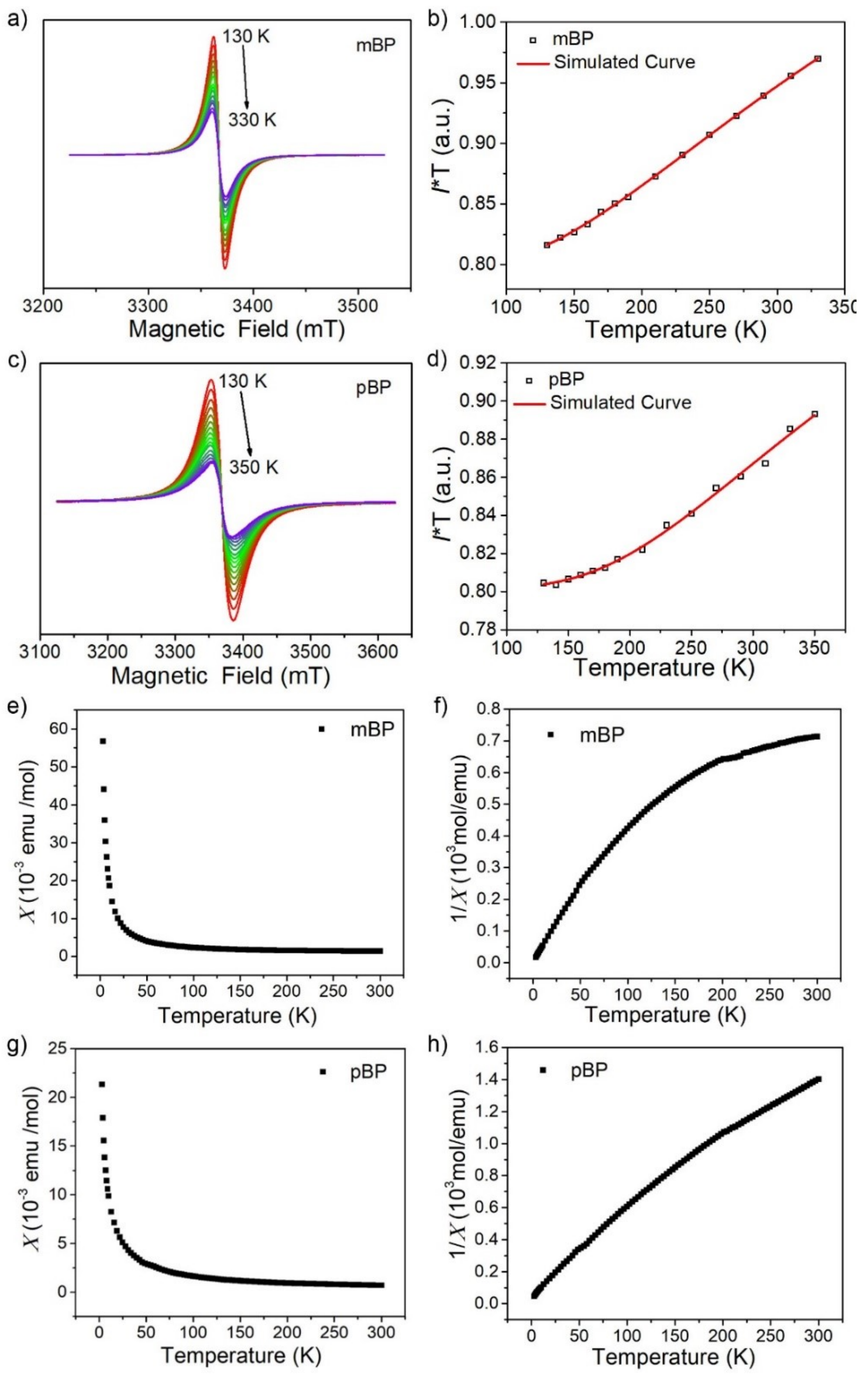

Figure S1. Fitted *I**T–T curve for **mBP** (a, b) and **pBP** (c, d) based on the VT ESR data measured in powder; I: integrated intensity; T: temperature. And χ vs T and 1/χ vs T curves for **mBP** (e, f) and **pBP** (g, h).

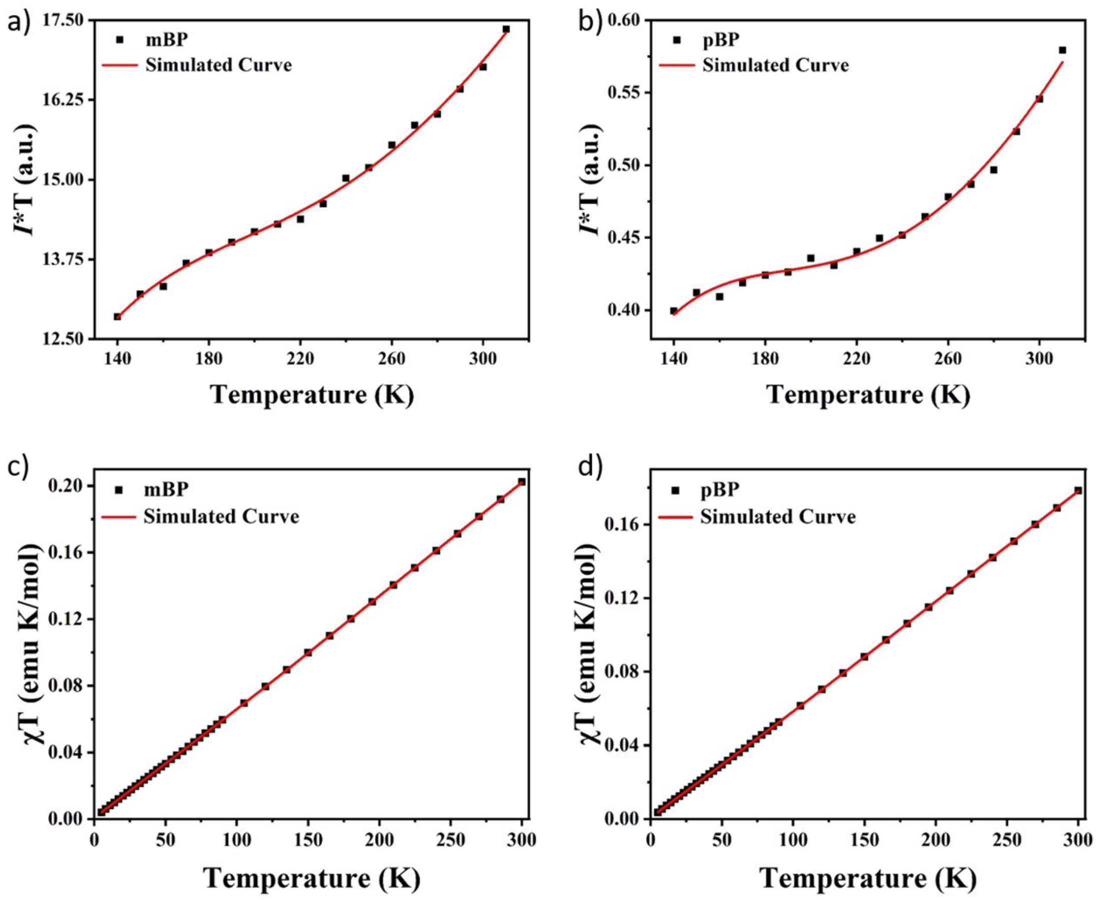

Figure S2. Fitted *I**T–T curve for **mBP** (a) and **pBP** (b) based on the VT-ESR data measured by dilute solid solution (benzoquinone glassy matrices with conc. 0.1 mM).; I: integrated intensity; T: temperature. And Magnetic susceptibilities (χT) versus T curve from the SQUID measurements for **mBP** (c) and **pBP** (d) in dilute solid solution (benzoquinone glassy matrices with conc. 40 mM).

The magnetic properties of both compounds were measured in dilute solid solution (benzoquinone glassy matrices for ESR and SQUID) as shown in Figure S2. We used benzoquinone instead of polystyrene in SQUID measurement due to keeping the same conditions for both experiments. From the fitting of this curve with the Bleaney-Bowers equation, the energy difference between the singlet (E_S) and triplet states (E_T), ΔE_{ST}, was estimated to -1.10/-1.08 and -1.07/-1.07 kcal/mol of **mBP** and **pBP** for ESR/SQUID, respectively. In comparsion with powder result, the ΔE_{ST}

values do not change much and also suggest the ground state of these moleucles are singlet groud state.

4. Low temperature absorption spectra

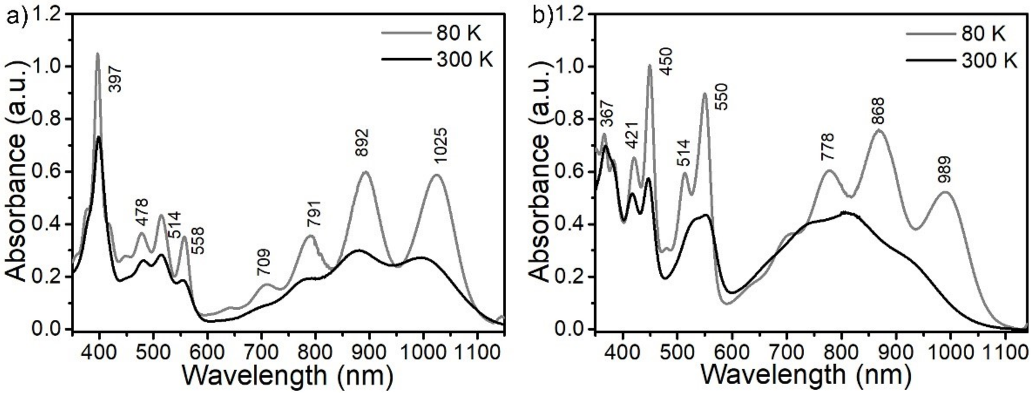

Figure S3. UV-Vis-NIR electronic absorption spectra of **mBP** (a) and **pBP** (b) in 2-MeTHF as a function of the temperature.

5. Electrochemical properties

Cyclic voltammetry was performed to investigate electrochemical properties of **mBP** and **pBP** in dichloromethane (DCM) (Figure S4). Both exhibited three reversible reduction and oxidation waves with half-wave potential displayed in Table S1. The energy levels of the HOMO and LUMO were determined to be -4.37 and -3.51 eV for **mBP**, -4.34 and -3.62 eV for **pBP**, respectively, from the onset potentials of the oxidation and reduction waves. Thus, a low electrochemical energy gap (E_g^{EC}) of 0.86 and 0.72 eV was determined for **mBP** and **pBP**, which was consistent with their optical energy gaps (E_g^{Opt} = 0.95 and1.09 eV). Moreover, the thermostability of **mBP** and **pBP** was investigate by thermo-gravimetric analysis (TGA), and as shown in Figure S5d, **mBP** is more stable than **pBP**, as the mass of **mBP** and **pBP** began to loss at 140 and 190 °C, respectively.

Table S1. Photophysical and electrochemical data of **mBP** and **pBP**.

	$E_{1/2}^{ox}{}_1$ (V)	$E_{1/2}^{ox}{}_2$ (V)	$E_{1/2}^{ox}{}_3$ (V)	$E_{1/2}^{red}{}_1$ (V)	$E_{1/2}^{red}{}_2$ (V)	$E_{1/2}^{red}{}_3$ (V)	HOMO (eV)	LUMO (eV)	E_g^{EC} (eV)	E_g^{opt} (eV)
pBP	-0.56	-0.18	0.13	-1.15	-1.33	-1.56	-4.34	-3.62	0.72	1.09
mBP	-0.64	-0.50	-0.11	-1.22	-1.54	-1.74	-4.37	-3.51	0.86	0.95

Definitions: λ_{abs}, absorption maxima measured in DCM; ε_{max} is molar extinction coefficient measured at absorption maximum; $E_{1/2}^{ox}$ and $E_{1/2}^{red}$ are the half-wave potentials for respective oxidation and reduction waves with Fc/Fc^+ as reference. HOMO and LUMO energy levels were calculated according to the equations HOMO = − (4.8 + E_{ox}^{onset}) eV and LUMO = − (4.8 +E_{red}^{onset}) eV, where E_{ox}^{onset} and E_{red}^{onset} are the onset potentials of the first oxidative and reductive redox wave, respectively. E_g^{EC} is the electrochemical energy gap derived from LUMO−HOMO, and E_g^{opt} is the optical energy gap derived from the lowest energy absorption onset in the absorption spectrum.

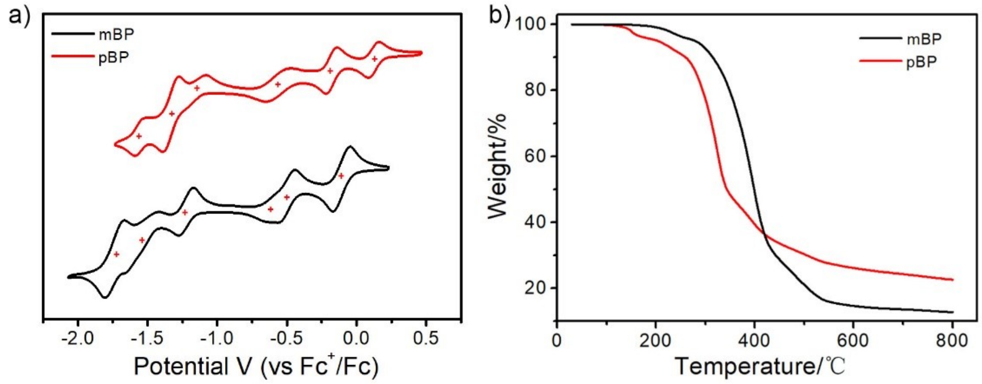

Figure S4. a) Cyclic voltammogram of **mBP** and **pBP** in DCM with 0.1 M Bu_4NPF_6 as supporting electrolyte, Ag/AgCl as the reference electrode, Au disk as the working electrode, Pt wire as the counter electrode, and the scan rate at 50 mV/s; b) Thermogravimetric analysis of **mBP** and **pBP**.

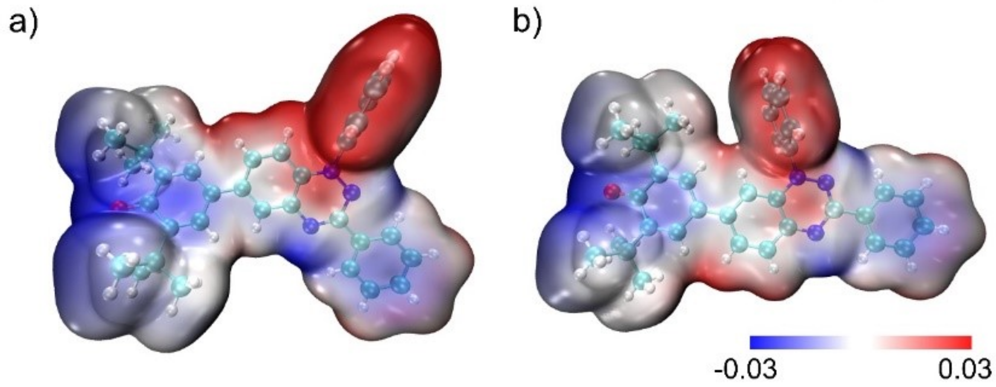

Figure S5. Electrostatic potential surface of **mBP** (e) and **pBP** (f) based on single crystal structure at the (u)m062x/6- 311(d,p) level.

6. NMR, Masspectroscopy and Fourier transform infrared (FT-IR) spectra

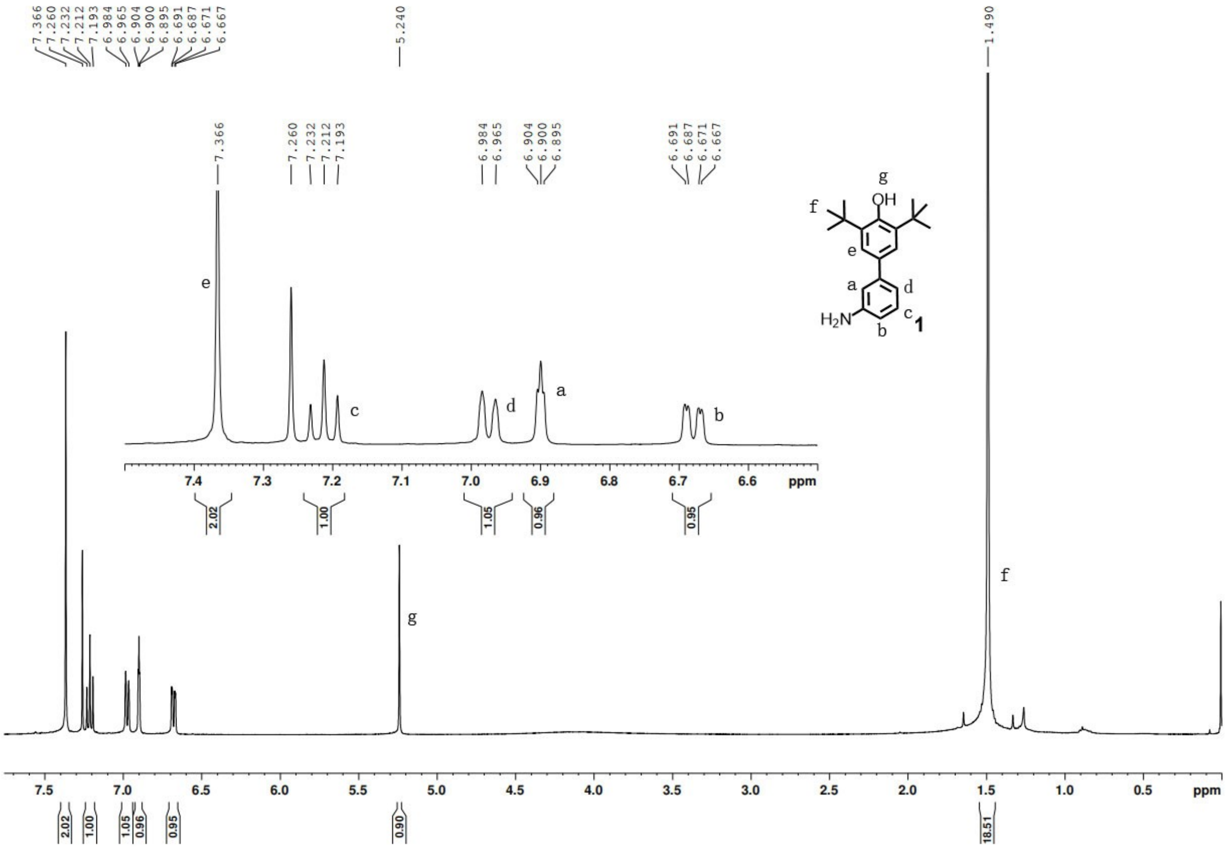

Figure S6. ^{1}H NMR spectrum of **1** (400 MHz, $CDCl_3$).

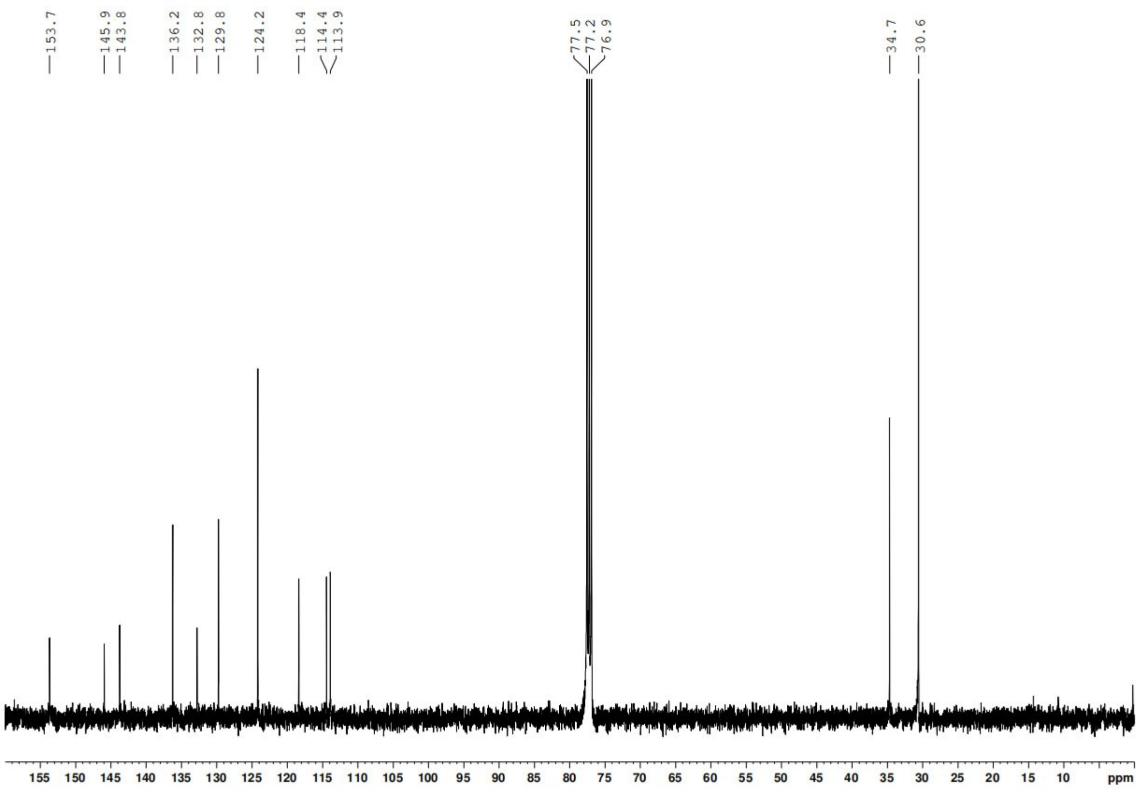

Figure S7. ^{13}C NMR spectrum of **1** (100 MHz, $CDCl_3$)

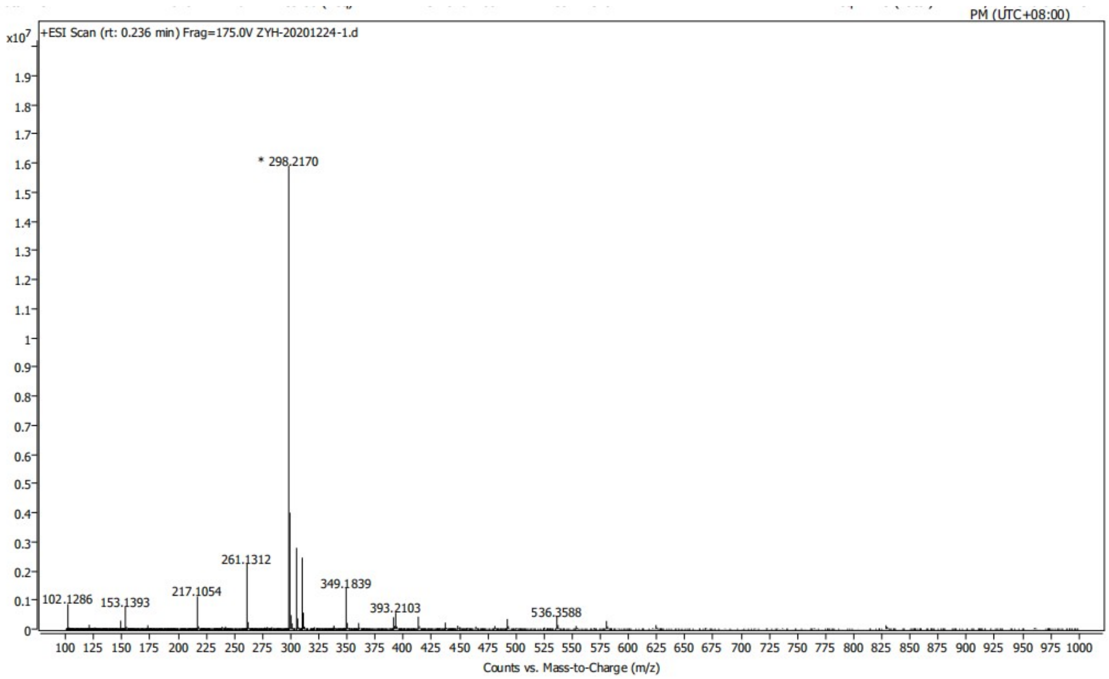

Figure S8. HR-Mass spectrum of **1**.

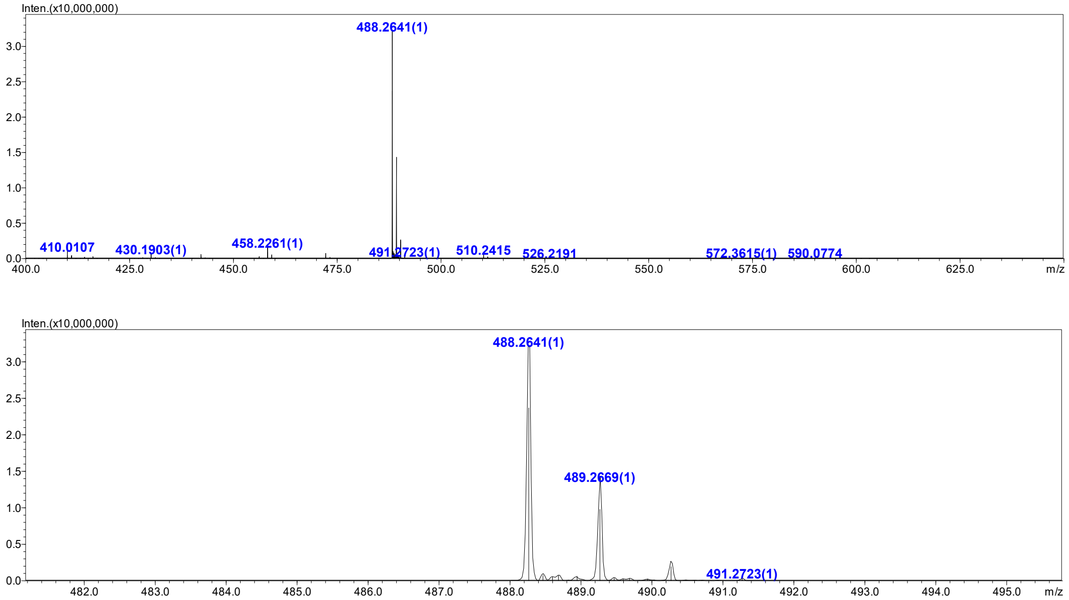

Figure S9. HR-Mass spectrum of **mBP**.

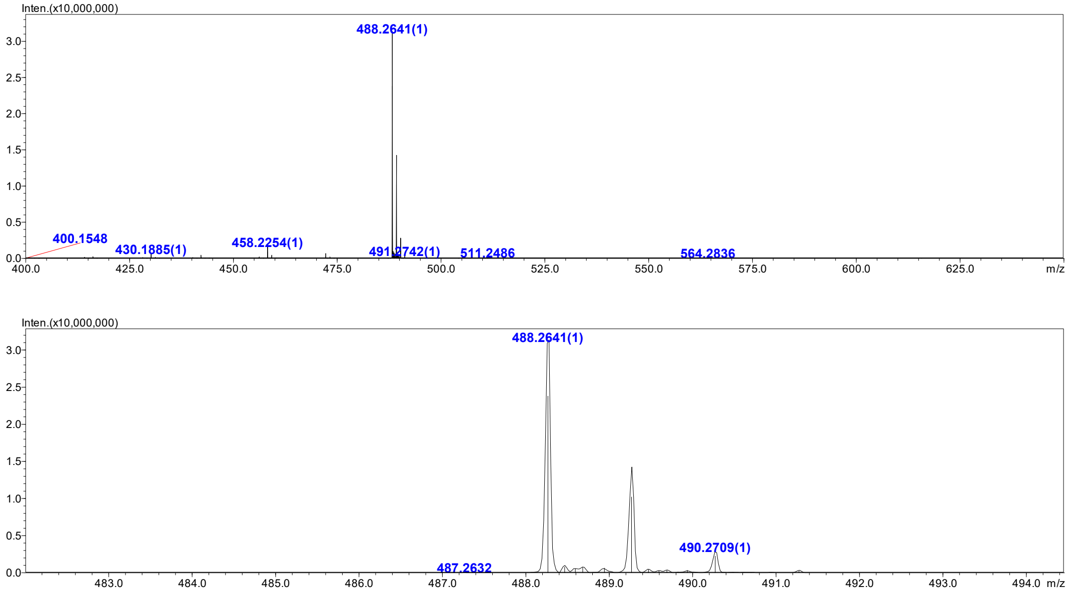

Figure S10. HR-Mass spectrum of **pBP**.

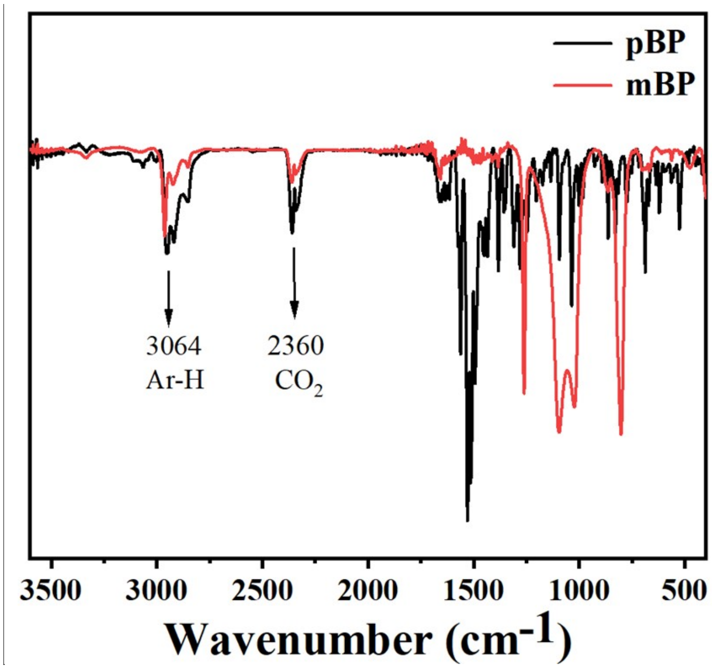

Figure S11. FT-IR spectra of **pBP** and **mBP**.

7. X-ray single crystal data

Table S2. Sample and crystal data for **mBP** (CCDC Number: 2049604)

Identification code	ABCZ7170	
Chemical formula	$C_{33}H_{33}N_3O$	
Formula weight	487.62 g/mol	
Temperature	184(2) K	
Wavelength	1.54178 Å	
Crystal size	0.100 x 0.100 x 0.100 mm	
Crystal system	monoclinic	
Space group	P 1 21/c 1	
Unit cell dimensions	a = 15.527(3) Å	α = 90°
	b = 10.1852(18) Å	β = 108.352(7)°
	c = 18.020(3) Å	γ = 90°
Volume	2704.8(8) $Å^3$	
Z	4	
Density (calculated)	1.197 g/cm^3	
Absorption coefficient	0.564 mm^{-1}	
F(000)	1040	
Final R indices	3530 data; I>2σ(I)	R1 = 0.0487, wR2 = 0.1076
	all data	R1 = 0.0693, wR2 = 0.1212
Weighting scheme	$w=1/[\sigma^2(F_o^2)+(0.0429P)^2+0.7931P]$ where $P=(F_o^2+2F_c^2)/3$	
Absolute structure parameter	0.00(3)	
Largest diff. peak and hole	0.195 and -0.183 eÅ^{-3}	
R.M.S. deviation from mean	0.041 eÅ^{-3}	

Table S3. Data collection and structure refinement for **mBP** (CCDC Number: 2049604)

Theta range for data collection	3.00 to 66.52°
Index ranges	-18<=h<=18, -12<=k<=12, -19<=l<=21
Reflections collected	16365
Independent reflections	4757 [R(int) = 0.0500]
Coverage of independent reflections	99.5%
Absorption correction	Multi-Scan
Max. and min. transmission	0.9500 and 0.8000
Structure solution technique	direct methods
Structure solution program	XT, VERSION 2018/2

Refinement method	Full-matrix least-squares on F^2	
Refinement program	SHELXL-2018/3 (Sheldrick, 2018)	
Function minimized	Σ $w(F_o^2 - F_c^2)^2$	
Data / restraints / parameters	4757 / 0 / 340	
Goodness-of-fit on F2	1.045	
Final R indices	3530 data; I>2σ(I)	R1 = 0.0487, wR2 = 0.1076
	all data	R1 = 0.0693, wR2 = 0.1212
Weighting scheme	$w=1/[\sigma^2(F_o^2)+(0.0429P)^2+0.7931P]$ where $P=(F_o^2+2F_c^2)/3$	
Absolute structure parameter	0.00(3)	
Largest diff. peak and hole	0.195 and -0.183 eÅ^{-3}	
R.M.S. deviation from mean	0.041 eÅ^{-3}	

Table S4. Sample and crystal data for **pBP** (CCDC Number: 2049603)

Identification code	ABCZ7170	
Chemical formula	$C_{33}H_{33}N_3O$	
Formula weight	487.62 g/mol	
Temperature	184(2) K	
Wavelength	1.54178 Å	
Crystal size	0.050 x 0.100 x 0.200 mm	
Crystal system	orthorhombic	
Space group	P n m a	
Unit cell dimensions	a = 27.4526(15) Å	α = 90°
	b = 6.8462(4) Å	β = 90°
	c = 18.7535(12) Å	γ = 90°
Volume	3524.6(4) Å^3	
Z	4	
Density (calculated)	0.919 g/cm^3	
Absorption coefficient	0.433 mm^{-1}	
F(000)	1040	
R.M.S. deviation from mean	0.034 eÅ^{-3}	

Table S5. Data collection and structure refinement for **pBP** (CCDC Number: 2049603)

Theta range for data collection	3.22 to 66.67°
Index ranges	-26<=h<=32, -8<=k<=8, -22<=l<=15
Reflections collected	13669
Independent reflections	3380 [R(int) = 0.0489]
Coverage of independent reflections	99.1%
Absorption correction	Multi-Scan

Max. and min. transmission	0.7528 and 0.5892	
Structure solution technique	direct methods	
Structure solution program	XT, VERSION 2018/2	
Refinement method	Full-matrix least-squares on F2	
Refinement program	SHELXL-2018/3 (Sheldrick, 2018)	
Function minimized	Σ w(F_o^2 - F_c^2)^2	
Data / restraints / parameters	3380 / 0 / 211	
Goodness-of-fit on F2	1.069	
Δ/σmax	0.003	
Final R indices	2234 data; I>2σ(I)	R1 = 0.0558, wR2 = 0.1653
	all data	R1 = 0.0795, wR2 = 0.1828
Weighting scheme	w=1/[σ^2(F_o^2)+(0.0934P)^2+0.1813P] where P=(F_o^2+2F_c^2)/3	
Absolute structure parameter	0.00(3)	
Largest diff. peak and hole	0.151 and -0.184 eÅ^{-3}	
R.M.S. deviation from mean	0.034 eÅ^{-3}	

8. References

1. W. J. Hehre, R. Ditchfield and J. A. Pople, *J. Chem. Phys.*, **1972**, *56*, 2257-2261.
2. M. Nakano, R. Kishi, T. Nitta, T. Kubo, K. Nakasuji, K. Kamada, K. Ohta, B. Champagne, E. Botek and K. Yamaguchi, *J. Phys. Chem. A*, **2005**, *109*, 885-891.
3. R. A. Gaussian 16, M. J. Frisch, G. W. Trucks, H. B. Schlegel, G. E. Scuseria, M. A. Robb, J. R. Cheeseman, G. Scalmani, V. Barone, G. A. Petersson, H. Nakatsuji, X. Li, M. Caricato, A. V. Marenich, J. Bloino, B. G. Janesko, R. Gomperts, B. Mennucci, H. P. Hratchian, J. V. Ortiz, A. F. Izmaylov, J. L. Sonnenberg, D. Williams-Young, F. Ding, F. Lipparini, F. Egidi, J. Goings, B. Peng, A. Petrone, T. Henderson, D. Ranasinghe, V. G. Zakrzewski, J. Gao, N. Rega, G. Zheng, W. Liang, M. Hada, M. Ehara, K. Toyota, R. Fukuda, J. Hasegawa, M. Ishida, T. Nakajima, Y. Honda, O. Kitao, H. Nakai, T. Vreven, K. Throssell, J. A. Montgomery, Jr., J. E. Peralta, F. Ogliaro, M. J. Bearpark, J. J. Heyd, E. N. Brothers, K. N. Kudin, V. N. Staroverov, T. A. Keith, R. Kobayashi, J. Normand, K. Raghavachari, A. P. Rendell, J. C. Burant, S. S. Iyengar, J. Tomasi, M. Cossi, J. M. Millam, M. Klene, C. Adamo, R. Cammi, J. W. Ochterski, R. L. Martin, K. Morokuma, O. Farkas, J. B. Foresman, and D. J. Fox, Gaussian, Inc. Wallingford CT. **2016**.
4. Y. Zhao and D. G. Truhlar, The M06 suite of density functionals for main group thermochemistry, thermochemical kinetics, noncovalent interactions, excited states, and transition elements: two new functionals and systematic testing of four M06 functionals and 12 other functionals, *Theoretical Chemistry Accounts*, **2008**, *119*, 525-525.
5. W. J. Hehre, R. Ditchfield and J. A. Pople, Self—Consistent Molecular Orbital Methods. XII. Further Extensions of Gaussian—Type Basis Sets for Use in Molecular Orbital Studies of Organic Molecules *Journal of Chemical Physics*, **1972**, *56*, 2257-2261.
6. M. Francl, W. J. Pietro, W. J. Hehre, J. S. Binkley, M. S. Gordon, D. J. Defrees and J. A. Pople, Self-consistent molecular orbital methods 25. Supplementary functions for Gaussian basis sets, *Journal of Chemical Physics*, **1982**, *77*, 3654-3665.
7. M. J. Frisch, J. A. Pople and J. S. Binkley, Self-consistent molecular orbital methods 25. Supplementary functions for Gaussian basis sets *Journal of Chemical Physics*, **1984**, *80*, 3265-3269.
8. T. Lu and F. Chen, Multiwfn: a multifunctional wavefunction analyzer *J Comput Chem*, **2012**, *33*, 580-592.

9. W. Humphrey, A. Dalke and K. Schulten, VMD: visual molecular dynamics *J Mol Graph*, **1996**, *14*, 33-38.
10. Nakano, M.; Kishi, R.; Nitta, T.; Kubo, T.; Nakasuji, K.; Kamada, K.; Ohta, K.; Champagne, B.; Botek, E.; Yamaguchi, K. Second Hyperpolarizability (γ) of Singlet Diradical System: Dependence of γ on the Diradical Character. J. Phys. Chem. A **2005**, *109*, 885-891.
11. Nakano, M.; Takebe, A.; Kishi, R.; Fukui, H.; Minami, T.; Kubota, K.; Takahashi, H.; Kubo, T.; Kamada, K.; Ohta, K. Intermolecular interaction effects on the second hyperpolarizability of open-shell singlet diphenalenyl radical dimer. Chem. Phys. Lett. 2008, 454, 97-104.
12. S. Stoll, A. Schweiger, *J. Magn. Reson.*, **2006**, *178*, 42.

9. Coordinates for calculated geometries (Å)

mBP

O	-6.23857044	-0.59861033	0.32231481
N	2.44321919	-1.43689182	-0.44579123
N	3.28426178	1.12314100	0.00486156
N	4.22115517	0.13642569	-0.06413408
C	-6.28880466	2.44234777	0.57415115
H	-6.79071938	1.67448245	0.91983532
H	-6.90836771	3.17505228	0.37626270
H	-5.63871480	2.73637160	1.24612248
C	-5.54992209	2.03494158	-0.70930046
C	-4.53551719	0.91776568	-0.40761911
C	-3.19227465	1.11084671	-0.57201048
H	-2.89295288	1.96276808	-0.86716251
C	-2.22134063	0.10542401	-0.32493756
C	-0.79925925	0.36446233	-0.39173874
C	0.15701052	-0.65517721	-0.48753441
H	-0.13861579	-1.54598447	-0.63459033
C	1.51610630	-0.41468511	-0.37623650
C	3.72162113	-1.09375068	-0.30362488

C	4.75071432	-2.16221492	-0.38286159
C	4.36923335	-3.48351149	-0.59155897
H	3.45172646	-3.69505203	-0.71794960
C	5.31478112	-4.49177582	-0.61640893
H	5.04241002	-5.39069382	-0.75768121
C	6.65332618	-4.20092818	-0.43782618
H	7.29796806	-4.89826120	-0.45344445
C	1.95437776	0.93035965	-0.15876624
C	1.01603110	1.97929462	-0.12309163
H	1.30524004	2.87749961	-0.01365247
C	-0.30614418	1.69584115	-0.24655535
H	-0.92884991	2.41335366	-0.23577482
C	-2.69384422	-1.16380656	0.10153680
H	-2.06086463	-1.85969485	0.23451475
C	-4.01601771	-1.43483716	0.33038009
C	-4.99488397	-0.37593328	0.08935136
C	-6.56704632	1.55972848	-1.76329925
H	-6.11946090	1.46438109	-2.62956971
H	-7.28996208	2.21688787	-1.83910810
H	-6.93921610	0.69479779	-1.49084901
C	-4.87300930	3.29379121	-1.25096157
H	-4.25929158	3.65132356	-0.57594660
H	-5.55425494	3.96636207	-1.46181160
H	-4.37163042	3.07159962	-2.06335554
C	-4.47034856	-2.80115956	0.87572140
C	-5.43801083	-3.47024587	-0.12037997
H	-6.22126481	-2.89600760	-0.25078962
H	-5.72455561	-4.33755311	0.23551532
H	-4.98361306	-3.60330457	-0.97812113
C	-5.15590532	-2.62106970	2.23664232
H	-4.52712104	-2.21244298	2.86751486

H	-5.43979977	-3.49466116	2.57766400
H	-5.93878746	-2.04032175	2.13368109
C	-3.28021566	-3.74827095	1.08696357
H	-2.81253801	-3.88058684	0.23622124
H	-3.60481835	-4.61164400	1.41900794
H	-2.66404154	-3.35785259	1.74143526
C	3.83916742	2.41800808	0.34400752
C	4.49067199	3.15319011	-0.62101182
H	4.54802301	2.83420470	-1.51411281
C	5.06157531	4.36703184	-0.26888511
H	5.51725814	4.88588201	-0.92077823
C	4.96639686	4.81768070	1.02836475
H	5.36175258	5.64733086	1.26867717
C	4.30111284	4.07387264	1.98427922
H	4.23814924	4.39554036	2.87599310
C	3.72489430	2.85887775	1.64507068
H	3.26230535	2.34226269	2.29443789
C	6.10597567	-1.86940914	-0.21628144
H	6.38186048	-0.96941741	-0.08836133
C	7.05549661	-2.88712957	-0.23635483
H	7.97516888	-2.68381797	-0.11223929

pBP

N	4.01698613	0.49337813	0.00001780
O	-5.98843063	0.06139688	0.00000296
C	8.58368118	-1.53646258	-0.00004447
H	9.51789847	-1.70829031	-0.00004966
C	8.12264301	-0.24196138	-0.00000440
H	8.73914774	0.48072579	0.00001809
N	2.69358911	0.74699011	0.00002547
N	3.53639102	-1.85695233	-0.00005514

C	6.75766597	0.01169377	0.00000327
H	6.44621981	0.90916537	0.00003105
C	5.84329218	-1.03250075	-0.00002924
C	6.32959179	-2.33652889	-0.00006960
H	5.72263702	-3.06733131	-0.00009235
C	4.39155490	-0.77172089	-0.00002137
C	7.69859863	-2.57466081	-0.00007679
H	8.02111152	-3.46828315	-0.00010445
C	1.72253933	-0.24716362	-0.00000549
C	2.24125778	-1.58554431	-0.00004691
C	0.38267461	0.01322736	0.00000239
H	0.08363543	0.91491407	0.00003030
C	-0.56790799	-1.03059190	-0.00003011
C	-0.04533885	-2.36476519	-0.00007140
H	-0.65742342	-3.09137181	-0.00009402
C	1.27390897	-2.63630575	-0.00007963
H	1.56540380	-3.54039516	-0.00010762
C	-1.96796247	-0.76884333	-0.00002220
C	-2.46191507	0.56778437	0.00001917
H	-1.82880128	1.27598606	0.00004122
C	-3.77656318	0.89296804	0.00002906
C	-4.76808399	-0.19768531	-0.00000490
C	-4.28009074	-1.58620074	-0.00004788
C	-2.94267389	-1.81097449	-0.00005465
H	-2.63827519	-2.71105637	-0.00008251
C	-5.29649212	-2.73973445	-0.00008379
C	-4.58229628	-4.10397248	-0.00012598
H	-4.01895121	-4.17782091	0.79847187
H	-5.24926294	-4.82196018	0.00315166
C	2.30308812	2.13017633	0.00006830
C	2.07151241	2.76932236	-1.19931200

H	2.21561613	2.31534345	-2.02122612
C	1.62731314	4.07826183	-1.19177149
H	1.47309520	4.53255251	-2.01175748
C	1.40920660	4.72134642	0.00014851
H	1.10403335	5.62106764	0.00027636
C	-3.06024086	3.32395129	0.00010454
H	-2.51119609	3.16707411	-0.79630030
H	-3.39051450	4.24649531	-0.00716691
C	-4.24637417	2.35766731	0.00007441
C	-5.06695458	2.64527322	-1.26021689
H	-5.33200959	3.58870991	-1.26948767
H	-5.86805826	2.08084848	-1.26463450
H	-4.52590594	2.45042017	-2.05402291
C	-6.16524119	-2.65905397	-1.25838150
H	-6.78950720	-3.41448153	-1.27320502
H	-5.59314465	-2.69239529	-2.05308251
H	-6.66956547	-1.81871919	-1.25355552
H	-4.01895098	-4.17777140	-0.79862825
C	2.07151206	2.76924799	1.19958817
H	2.21561555	2.31521811	2.02147417
C	1.62731279	4.07818792	1.19212869
H	1.47309462	4.53242775	2.01214281
H	-2.51119632	3.16702473	0.79659981
C	-5.06695494	2.64519507	1.26048329
H	-5.33200996	3.58863118	1.26981250
H	-5.86805863	2.08077006	1.26486568
H	-4.52590654	2.45029279	2.05427738
C	-6.16524156	-2.65913200	1.25831867
H	-6.78950757	-3.41456048	1.27309517
H	-5.59314523	-2.69252260	2.05301778
H	-6.66956584	-1.81879692	1.25354466
